# A New Method to Improve the Detection of Co-Seismic Ionospheric Disturbances using Sequential Measurement Combination

**DOI:** 10.3390/s19132948

**Published:** 2019-07-04

**Authors:** Seonho Kang, Junesol Song, Deokhwa Han, Bugyeom Kim, Hyoungmin So, Kap-jin Kim, Changdon Kee

**Affiliations:** 1 Mechanical and Aerospace Engineering, and the SNU-IAMD, Seoul National University, Seoul 08826, Korea; 2 Ecole Nationale de l’Aviation Civile (ENAC), Toulouse 31400, France; 3 Agency for Defense Development, Daejeon 305-600, Korea

**Keywords:** co-seismic ionospheric disturbances, earthquake, signal-to-noise ratio, ionosphere

## Abstract

Earthquakes generate energy that propagates into the ionosphere and incurs co-seismic ionospheric disturbances (CIDs), which can be observed in ionospheric delay measurements. In most cases, the CID has a weak signal strength, because the energy in the atmosphere transferred from the earthquake dissipates as it travels toward the ionosphere. It is particularly hard to observe at reference stations that are located far from the epicenter. As the number of Global Navigation Satellite System stations and their positions are restricted, it is important to employ weak CID data in the analysis by improving the detection performance of CIDs. In this study, we suggest a new method of detecting CIDs, which mainly uses a sequential measurement combination of the carrier phase-based ionospheric delay data, with a 1-second interval. The proposed method’s performance was compared with conventional methods, including band-pass filters and a representative time-derivative method, using data from the 2011 Tohoku earthquake. As a result, the maximum CID-to-noise ratio can be increased by a maximum of 13% when the proposed method is used, and consequently, the detection performance of the CID can be improved.

## 1. Introduction

Earthquakes can result in disturbed electron densities in the ionosphere, which are called co-seismic ionospheric disturbances (CIDs). When an earthquake occurs, its energy is transferred to the atmosphere by solid earth–atmosphere coupling. This waveform energy travels through the atmosphere, and it is amplified, due to the combined effect of an exponentially decreasing air density and conserved kinematic energy. It eventually arrives in the ionosphere, and affects the electron density by momentum transfer between neutral particles and electrons [1,2,3]. Consequently, a disturbed electron density is observed shortly after earthquake events.

Global Navigation Satellite System (GNSS) measurements can be used to estimate the electron density in the ionosphere. The ionospheric delay error in the GNSS signal is proportional to the total electron content (TEC), which are the integrated electrons along the signal path. As TEC, in turn, is inversely proportional to the square of the signal frequency, it can be estimated with a linear combination of L1 and L2 frequency signals [4]. 

The sources of the CIDs can be classified into three types of waves, the first of which is a Rayleigh wave. A Rayleigh wave is a ground wave that propagates from the epicenter when earthquakes occur, and its speed is around 3.5 km/s. The vertical movement of the ground surface induces acoustic waves. As the Rayleigh wave propagates through the ground surface, the acoustic wave is initiated from the ground, and it reaches the ionosphere after the Rayleigh wave [5]. Therefore, the same circular wave is observed in the ionosphere around 10 min after the signature of the ground Rayleigh wave. Here, the period of 10 min represents the time for the acoustic wave to travel from the ground to the height of the ionosphere, which is an altitude of 350 km. The second source of CIDs is a direct acoustic gravity wave (AGW), which is caused by an abrupt vertical movement of the ground at the epicenter. As this wave starts from the point source of the epicenter, it is attenuated rapidly. Previous studies have indicated that the CIDs from AGWs disappear at around 500–1000 km horizontal distance [6]. The final source of CIDs is tsunamis. Tsunami ocean surface waves induce gravity waves in the atmosphere, which reach the ionosphere and disturb the electron density. This type of CID has the same horizontal speed as a tsunami, which is around 200 m/s, and it is observed in the ionosphere ~30–40 min after the tsunami passes the same horizontal location on the water surface [6,7].

Among the three types of ionospheric disturbances, the Rayleigh wave CIDs are the first signatures to be detected, due to their fast speed. Also, as there are no ionospheric disturbances in the signal before the Rayleigh wave CID arrives, clear detection is possible, in comparison with other types of CIDs, where the ionosphere is already in a disturbed state. Therefore, in this study, we will focus on the CIDs caused by Rayleigh waves for the detection of ionospheric disturbances that are associated with earthquakes.

To allow for CID detection in ionospheric delay measurements, the normal trend, which includes the trend due to satellite geometry, local time, and the season, needs to be removed. There are several de-trending methods that are used to remove the nominal trend in ionospheric delay for when there is no ground impact, such as that from earthquakes. Band-pass filtering and high-pass filtering are commonly used to de-trend ionospheric delay measurements. The Rayleigh wave CID is known to have dominant frequencies of 3.7 mHz and 4.4 mHz [8], whereas the normal trend has a frequency of under 1 mHz. In order to de-trend the signal and to reduce noise, previous studies have used some designated passbands, such as 1–10 mHz [9], 1–8 mHz [7], 0.5–5 mHz [10], ~1.6–5.5 mHz [11], and ~3.3–5.5 mHz [12] with 30-s interval data. For 1-s interval data, Astafyeva et al. used the 0.8–333 mHz passband to remove the normal trend in the data [13].

The time derivative is another option for de-trending. Park et al. used the numerical third-order time derivative to remove the nominal trend [14,15], and Hernández-Pajares and Zhang used the time derivative with an extended time step [16,17].

Previous studies with both a band-pass filter and time derivative have focused on CID detection. However, few studies have investigated how to optimize the detection performance by increasing the relative magnitude of the CID-to-noise level. The signal-to-noise ratio (SNR) of CIDs is important when the magnitude of the CID is too small, either because the impacts of the earthquakes are small, or because the CID is detected far from the epicenter. As GNSS stations are limited in terms of CID detection coverage, the performance of weak signal detection needs to be improved, in order to employ all of the viable data.

To improve the SNR of the CID, we used the time derivative method. Time derivatives can be a useful tool for reducing noise in the signal, since its noise level can be numerically calculated with some assumptions [18]. In this study, we propose a new time derivative method to improve the SNR of CID. To maximize the detection performance, 1-s interval data will be used.

## 2. Methodology

Carrier phase L1 and L2 signals are denoted in Equation (1):

(1) $$\phi_{2}=d+B-b-I_{1}+T+\lambda_{1}N_{1}+\varepsilon_{1},\;\phi_{2}=d+B-b-I_{2}+T+\lambda_{2}N_{2}+\varepsilon_{2}.$$

The symbol of ϕ indicates a carrier phase, where the subscripts 1 and 2 indicate L1 and L2, respectively. The term d is the true range from the satellite to the receiver, B is the receiver clock bias, b is the satellite clock bias, I is the ionospheric delay error, T is the tropospheric delay error, λ is the wavelength of the carrier phase, N is the ambiguity, and ε is the noise of the carrier phase. In Equation (1), the multipath error is assumed to be zero. In addition, the satellite orbit error and inter-frequency bias are not considered here, because their time variations are small enough [19] such these can be neglected in the time derivatives of the carrier phase, which will be used for CID detection. As the ionospheric delay itself cannot be calculated directly, ionospheric combination or geometry-free combination is used, as shown in Equation (2):

(2) $$\phi_{I}=\frac{\phi_{1}-\phi_{2}}{\gamma -1}=I_{1}+\frac{\varepsilon_{1}-\varepsilon_{2}}{\gamma -1}+\frac{\lambda_{1}N_{1}-\lambda_{2}N_{2}}{\gamma -1}.$$

By taking the time derivative, the last term on the right side in Equation (2) disappears, since it is constant as long as there is no cycle slip error. By taking the higher level of the time derivatives, the *n*th-order time derivative of ionospheric combination can be shown in Equation (3):

(3) $$\phi_{I}{}^{\left(n\right)}=I_{1}{}^{\left(n\right)}+\frac{\varepsilon_{1}{}^{\left(n\right)}-\varepsilon_{2}{}^{\left(n\right)}}{\gamma -1}.$$

When an earthquake takes place, the CID signal is included in $I_{1}{}^{\left(n\right)}$. To detect the CID by using Equation (3) reliably, the effect of the noise term in Equation (3) should be minimized. The novel methods that are optimized to reduce the noise level of the time derivative will be addressed in the following sections.

### 2.1. Assumptions

In order to derive the time derivative of the ionospheric combination that is optimized to reduce the noise level, two assumptions were adopted in this paper. The first assumption is that the ionospheric delay changes linearly for a short time span. This assumption holds true, as the normal trend in the ionosphere has a very long period (>1000 s). As the ionospheric change by the normal trend is very slow, it is nearly linear within a short time span. The other assumption is that the ionospheric delay has Gaussian random noise. This assumption was adopted to simplify the computation of the standard deviation of the time derivative ionospheric delay.

### 2.2. De-Noising Methods Using Forward Numerical Differentiation

The simplest form of a time derivative is the forward difference [20]. However, in a 1-s interval data, the noise in the forward difference is so significantly large that even a sizable CID cannot be detected. The first method to reduce noise in the time derivative therefore uses the moving average [21,22]. We will call this the forward difference and the moving average (FDMA), and we consider it to be a conventional method. 

The ionospheric combination can be denoted via Equation (4), where f is the ionospheric combination, g is the true ionospheric delay, and ν is the Gaussian noise with $\nu \sim N(0,\sigma_{\nu }{}^{2})$:

(4) f=g+ν.

By taking the forward difference with a 1-s time-step, as in Equation (5) [20], and by using the moving average for 1 to (N − 1), the time derivative of the ionospheric combination can be computed as in Equation (6):

(5) $$f_{i}{}^{\prime }=f_{i+1}-f_{i},$$

(6) $$f^{\prime }{}_{FDMA}=\frac{1}{N-1}\sum_{i=1}^{N-1}f^{\prime }{}_{i}=\frac{f_{N}-f_{1}}{N-1}.$$

The subscript i indicates the epoch index. Then, through the linearity of Equation (4), the time derivative of noise in the ionospheric combination can be obtained via Equation (7):

(7) $$\nu^{\prime }{}_{FDMA}=\frac{\nu_{N}-\nu_{1}}{N-1}.$$

Here, $\nu^{\prime }{}_{FDMA}$ is the noise component in ionospheric combination with $\nu \sim N(0,\sigma_{\nu }{}^{2})$. The noise level of the FDMA can be calculated via Equation (8):

(8) $$\sigma_{\nu \prime_{FDMA}}=\frac{\sqrt{2}}{N-1}\sigma_{\nu }.$$

The second and more effective way of reducing noise is to use an extended time step for the time difference operation, and then to subsequently use the moving average. We will call this proposed method the time step and moving average (TSMA). The extension of the time step for a de-noising purpose has already been used by [17]; however, the moving average is additionally used to reduce the noise further in the TSMA. The forward difference with extended time step K can be expressed via Equation (9) [16,17]:

(9) $$f_{i}{}^{\prime }=\frac{f_{i+K}-f_{i}}{K}.$$

With the moving average applied for 1 to *M*, the time derivative of the ionospheric combination by the TSMA can be obtained via Equation (10):

(10) $$\begin{array}{ll} f\prime_{TSMA} & =\frac{1}{M}\sum_{j=i}^{i+M-1}\frac{f_{j+K}-f_{j}}{K}\\ & =\frac{-\left(f_{i}+\cdots f_{i+M-1}\right)+\left(f_{i+K}+\cdots +f_{i+K+M-1}\right)}{KM} \end{array}$$

As can be seen in Equation (10), the total length of the dataset is (K+M). In order to set the same data length for the derivative as that in the other methods, we can set N=(K+M). Then, Equation (10) becomes:

(11) $$\begin{array}{ll} f^{\prime }{}_{TSMA} & =\frac{-(f_{i}+\cdots f_{i+N-K-1})+(f_{i+K}+\cdots +f_{i+N-1})}{K(N-K)}\\ & =\frac{-(f_{i}+\cdots f_{i+K-1})+(f_{N-K}+\cdots +f_{i+N-1})}{K(N-K)}\;\;(K\leq \frac{N}{2}) \end{array}$$

The last term in Equation (11) can be obtained, since the elements in the numerator are correlated. The noise level can be calculated via Equation (12):

(12) $$\sigma_{\nu \prime_{TSMA}}=\frac{\sqrt{2K}}{K(N-K)}\sigma_{\nu }.$$

Based on the fact that the extreme values have zero gradient, the variable K that minimizes $\sigma_{\nu \prime_{TSMA}}$ can be computed as $\frac{N}{3}$, which implies that N needs to be a multiple of 3 for optimal performance. The noise level for this case is calculated with Equation (13). The same noise level holds true for the case of $K>\frac{N}{2}$ by symmetry of $f\prime_{TSMA}$:

(13) $$\sigma_{\nu \prime_{TSMA}}=\frac{3\sqrt{6}}{2N\sqrt{N}}\sigma_{\nu }.$$

For a data length of N, FDMA’s noise level is inversely proportional to N, while TSMA’s is inversely proportional to $N^{1.5}$. Therefore, TSMA has a better noise level compared to FDMA. Both methods, however, are not optimal combinations in terms of noise reduction, since the moving average is optimal only for independent datasets. Because the moving average was applied in both methods to the time-differenced data, which are correlated, optimization cannot be guaranteed. We therefore propose a time derivative method that ensures a minimum noise level for a given length of data.

### 2.3. The Minimum Noise Derivative (MND) Method

The derivation of the proposed method starts with a Taylor series expansion for N sequential epochs [20]:

(14) $$\begin{array}{l} f_{i+1}=f_{i}+hf_{i}^{\prime }+O(h^{2})\\ f_{i+2}=f_{i}+2hf_{i}^{\prime }+O(h^{2})\\ \vdots \\ f_{i+N-1}=f_{i}+(N-1)hf_{i}^{\prime }+O(h^{2}) \end{array}$$

According to the linearity assumption, the second- and higher-order terms, $O(h^{2})$, are assumed to be zero. Also, as this study deals with 1-s interval data only, the time step h equals 1. The time derivatives of the ionospheric combination, $f^{\prime }$, can be expressed as a linear combination of f using arbitrary constants, $a_{i}$’s, as demonstrated in the following procedure:

(15) $$\begin{array}{l} a_{1}f_{i+1}=a_{1}f_{i}+a_{1}f_{i}{}^{\prime }\\ a_{2}f_{i+2}=a_{2}f_{i}+2a_{2}f_{i}{}^{\prime }\\ \vdots \\ a_{N-1}f_{i+N-1}=a_{N-1}f_{i}+(N-1)a_{N-1}f_{i}{}^{\prime } \end{array}$$

(16) $$\begin{array}{cc} f_{i}^{\prime } & =\frac{-(\sum_{k=1}^{N-1}a_{k})f_{i}+\sum_{k=1}^{N-1}\left(a_{k}f_{i+k}\right)}{\sum_{k=1}^{N-1}ka_{k}}\\ & =c_{1}f_{i}+c_{2}f_{i+1}+\cdots +c_{N}f_{i+N-1}\\ & =(c_{1}g_{i}+c_{2}g_{i+1}+\cdots +c_{N}g_{i+N-1})+(c_{1}\nu_{i}+c_{2}\nu_{i+1}+\cdots +c_{N}\nu_{i+N-1})\\ & =g_{i}^{\prime }+\nu_{i}^{\prime } \end{array}$$

Here $g_{i}^{\prime }$ and $\nu_{i}^{\prime }$ represent the first derivative of ionospheric delay and noise at the *i*-th epoch. The standard deviation (STD) of $\nu_{i}^{\prime }$ is proportional to the root square sum of its coefficients. That is:

(17) $$\nu_{i}^{\prime }\sim N(0,\sigma_{\nu_{i}^{\prime }})\;\mathrm{where}\;\sigma_{\nu_{i}^{\prime }}=\sqrt{\sum_{k=1}^{N}c_{k}{}^{2}}\sigma_{\nu }.$$

In order to effectively detect the CID, noise level reduction is crucial. By using the fact that the extreme values have zero gradient, the equation constraints to determine the constant, $a_{i}$, can be derived as follows:

(18) $$\begin{array}{ll} J & =\sum c_{i}{}^{2}\\ & =\frac{{\left(\sum_{k=1}^{N-1}a_{k}\right)}^{2}+\sum_{k=1}^{N-1}{\left(a_{k}\right)}^{2}}{{\left(\sum_{k=1}^{N-1}ka_{k}\right)}^{2}}\\ & =\frac{A}{B^{2}} \end{array}$$

(19) $$\frac{\partial J}{\partial a_{i}}=\frac{B^{2}\frac{\partial A}{\partial a_{i}}-2AB\frac{\partial B}{\partial a_{i}}}{B^{4}}=0,$$

where,

(20) $$\begin{array}{l} \frac{\partial A}{\partial a_{i}}=2(\sum_{k=1}^{N-1}a_{k}+a_{i})\\ \frac{\partial B}{\partial a_{i}}=i \end{array}$$

From Equation (18), we see that the value of J does not change when divided by one value of $a_{i}$. Therefore, we set $a_{i}=1$ as a pivot. Using Equations (18) to (20), the following equation holds for i=2,3,⋯,(N−1):

(21) $$i(\sum_{k=1}^{N-1}a_{k}+a_{1})=\sum_{k=1}^{N-1}a_{k}+a_{i}.$$

Now that (N−1) equations are all set, the unknown variables $a_{1},a_{2},\cdots ,a_{N-1}$ are solvable. In the end, the sequential combination solution is as follows:

(22) $$\begin{array}{l} f_{i}^{\prime }=c_{1}f_{i}+c_{2}f_{i+1}+\cdots +c_{N}f_{i+N-1}\\ where\;\;c_{k}=\frac{-6(N-1)+12(k-1)}{\left(N-1)N(N+1\right)}\;\;(k=1,2,\cdots ,N) \end{array}$$

Then, the STD of $\nu_{i}^{\prime }$ can be computed via Equation (23):

(23) $$\sigma_{{\nu^{\prime }}_{MND}}=\sqrt{\frac{12}{\left(N-1)N(N+1\right)}}\sigma_{\nu }.$$

Table 1 shows time derivative equations and noise levels of the conventional and the proposed algorithms. Here N denotes the number of epochs for time derivative, *σ_ν_* the standard deviation of background noise in ionospheric delay measurements.

### 2.4. Noise Level Comparisons for FDMA, TSMA, and MND

**Figure 1:**
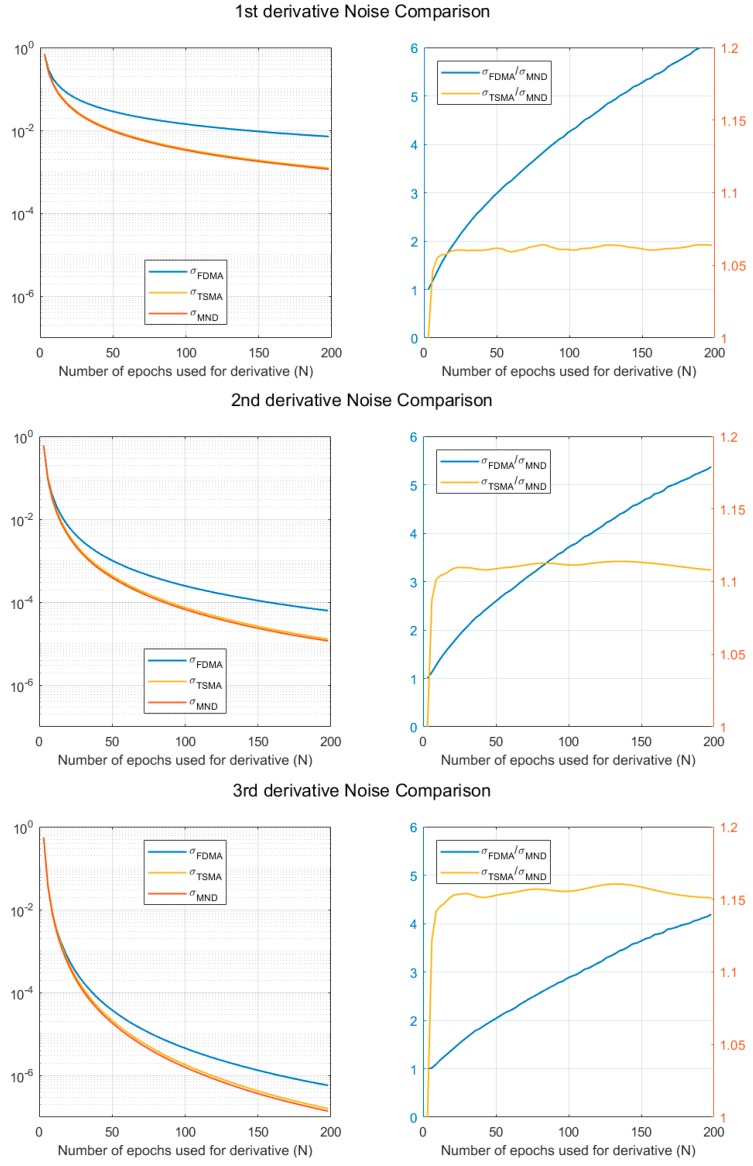
Noise level comparison for FDMA, TSMA, and MND.

Figure 1 shows the relative noise level comparison of FDMA, TSMA, and MND, according to the number of epochs for the time derivative, N. Here, $\sigma_{\nu }$ is assumed to be 1.

As can be seen from Figure 1, the noise levels of the TSMA and the MND are lower than that of the FDMA, as predicted from Equations (8), (13), and (23). Also, the MND has a better noise level compared to the TSMA. Specifically, for the third derivative, with N = 100, the TSMA has a 15% higher noise level compared to the MND. In short, it is reasonable to select the MND-based time derivative of ionospheric combination as the monitoring value for CID detection, in terms of noise reduction.

### 2.5. SNR and the Estimation of the Best N for CID Detection

Conventional SNR is defined as the ratio of signal variance to noise variance. However, the variance of Rayleigh-wave CID cannot be calculated in a simple way. For one thing, CID’s frequency of around a few mHz often coincides with that of the background noise and the ionospheric delay, in low elevation angle. Also, the Rayleigh wave CID has a fairly irregular duration time, in accordance with the distance from the epicenter, the magnitude of earthquake, the ionospheric condition, etc. As the exact span of the CID signal can hardly be specified for these reasons, it is possible that miscalculated signal variance could lead to a severely erroneous result with the conventional SNR. Therefore, we redefined the SNR as the ratio of the maximum absolute value of the CID signal to the noise STD. In this way, the detection performance of the CID could be analyzed in a safer way. In Equation (24), max(|CID|) indicates the maximum absolute value of the CID caused by a Rayleigh wave. This value is extracted from 10–30 min after an earthquake, to account for the time required for the impact of the earthquake to reach the ionosphere. The symbol of $\sigma_{noise}$ represents the noise STD in the region without the CID. This value is calculated by using 1-hr data that are collected just before the onset of the earthquake. This time span of the data is chosen to exclude the effect of the CID in the nominal STD, and to minimize the different effects of elevation angles on noise for nominal and disturbed data:

(24) $$SNR=\frac{\max(|CID|)}{\sigma_{noise}}.$$

While the noise level of the proposed time derivative method decreases monotonically, its SNR does not continuously increase with N. This is because the CID becomes nonlinear with a large N, and the nonlinearity curtails the peaks of the CID in the time derivative. Therefore, the best N, which maximizes the SNR of CID, needs to be determined for each algorithm.

**Figure 2:**
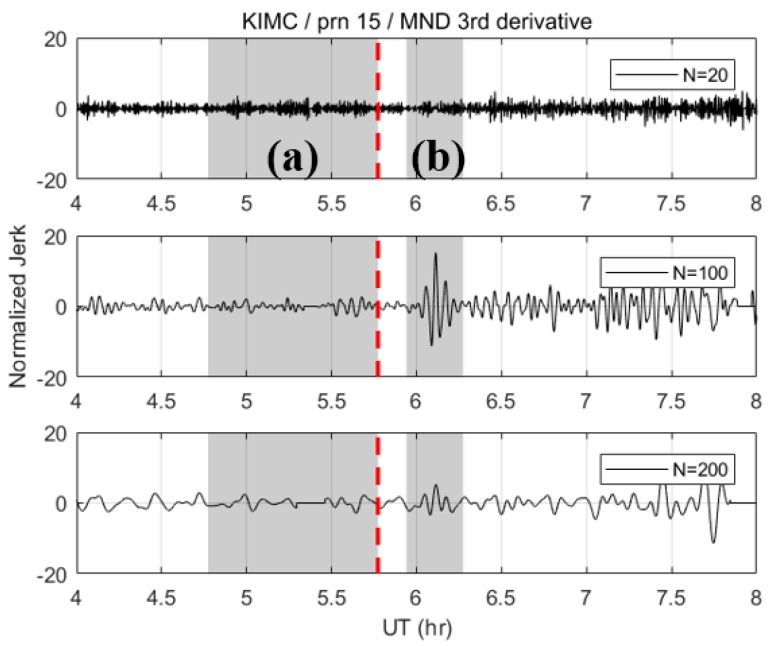
Normalized MND third derivatives with different N values: 20, 100, 200 (KIMC station, PRN 15).

The necessity of determining the best N can be demonstrated well by Figure 2, which shows the results of the MND with different values of N. The third-order derivative of the ionospheric combination was selected as the monitoring value to effectively remove the nominal trend in the data. Each dataset was normalized by the STD of shaded area (a), which corresponds to the 1-hr time span before the earthquake. The red vertical dashed line is the time of the earthquake occurrence. The shaded area (b) is the time span in which the CID is expected to arrive. This corresponds to ~10–30 min after the earthquake onset for the dataset that we used. The arrival time of the CID associated with the earthquake varies according to the distance between the epicenter and the point in the ionosphere through which the satellite signal passes. This point is called the ionospheric pierce point (IPP). As illustrated in Figure 2, N has a critical effect on the SNR and CID detection. To be more specific, when N=20 and N=200, the signatures of the CID hardly stand out from the noise. On the other hand, when N=100, the CID’s peaks are dominant in the time series. Finding the best N, therefore, is crucial to enhancing the detection performance of the CID.

**Figure 3:**
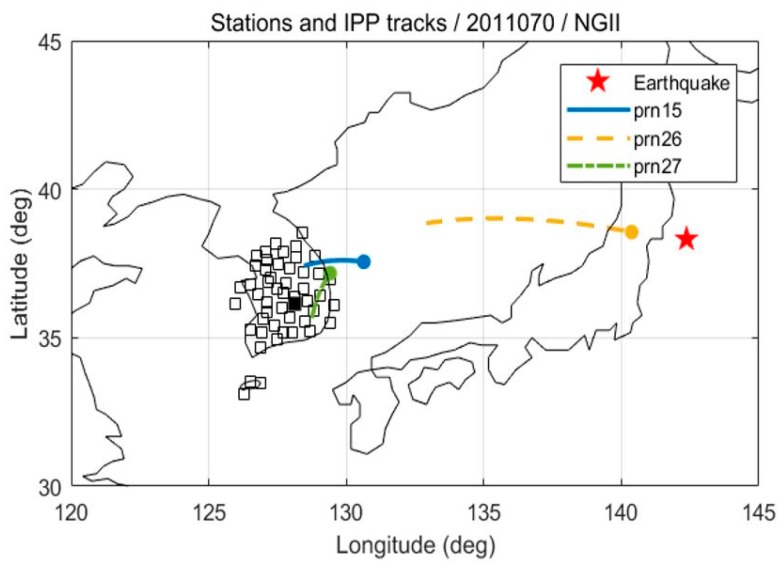
The location of the epicenter (red star), NGII stations (blank squares), and the IPP ground tracks for one hour from the earthquake outbreak, as observed at the KIMC station (filled square). The IPP moves toward the filled circles.

Due to the irregularity of the shape and duration of the CID, real data were used to estimate the best N. Data from the 2011 Tohoku earthquake, collected from National Geographic Information Institute (NGII) stations in Korea, were chosen. There were a total of 45 stations, and three GPS satellites were chosen for the proximity of their IPP tracks to the epicenter, as shown in Figure 3. 

**Figure 4:**
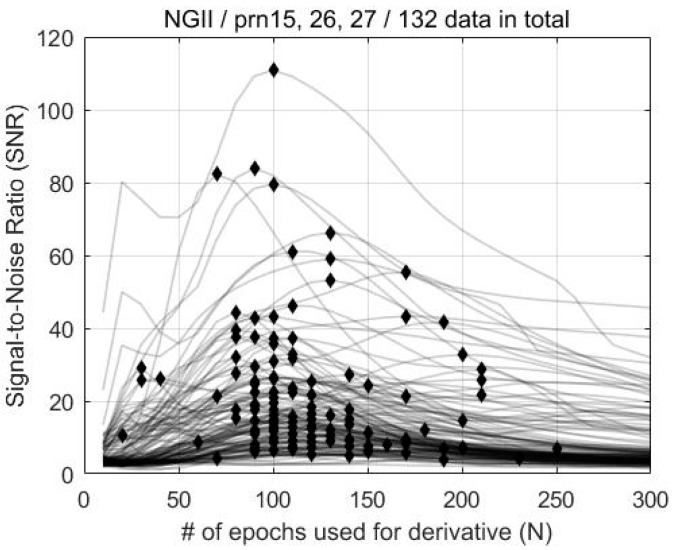
SNR, based on the MND method according to N, for the 2011 Tohoku Earthquake.

**Figure 5:**
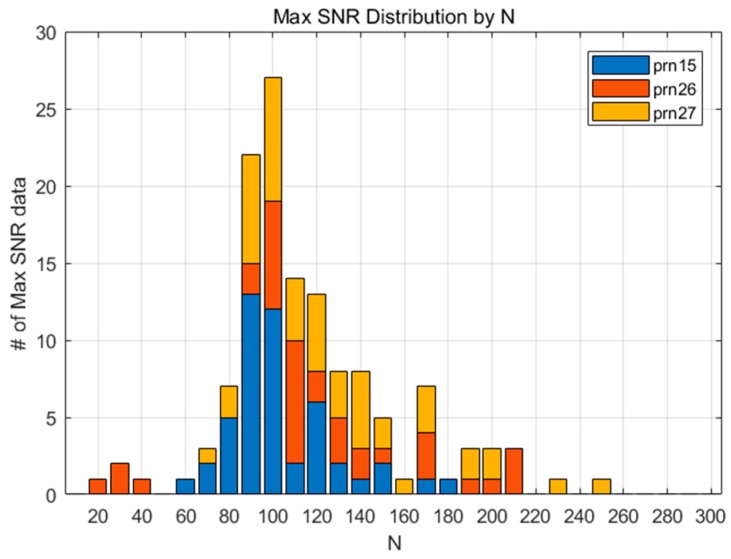
The maximum SNR distribution according to various values of N (MND)**.**

**Figure 6:**
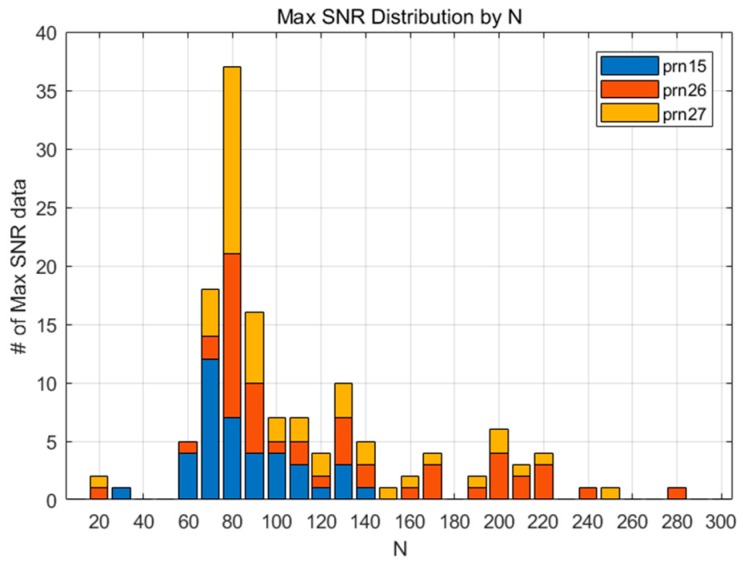
The maximum SNR distribution according to various values of N (FDMA)**.**

**Figure 7:**
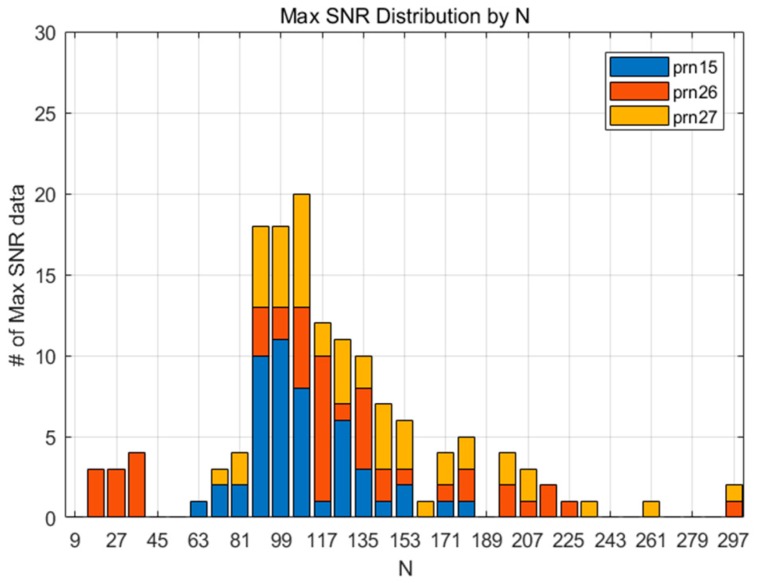
The maximum SNR distribution according to various values of N (TSMA)**.**

Figure 4 shows the SNR for 132 datasets according to N, and the pairs of the maximum SNR and corresponding N for each dataset are marked with a diamond. It can be inferred from the figure that the SNR variation of each measurement shows diverse shapes, even for the 2011 Tohoku Earthquake case alone. This means that a small number of CID samples cannot represent the overall characteristics of the phenomenon, which is the reason for why we adopted a statistical approach for finding the best N. As shown in Figure 5, the maximum SNRs are distributed around N=100. This means that the CID can be most successfully detected when N=100. Likewise, the maximum SNR for FDMA and TSMA are shown in Figure 6 and Figure 7. Based on these observations, the best N values for the MND, FDMA, and TSMA are determined, as in Table 2. The third-order derivative by FDMA has its best N at 80, while the TSMA has its best N at 108. The TSMA’s interval of N was set to be 9 instead of 10, because the TSMA algorithm requires a multiple of 3 for N.

### 2.6. Band-Pass Filter for 1-second Interval CID Data

**Figure 8:**
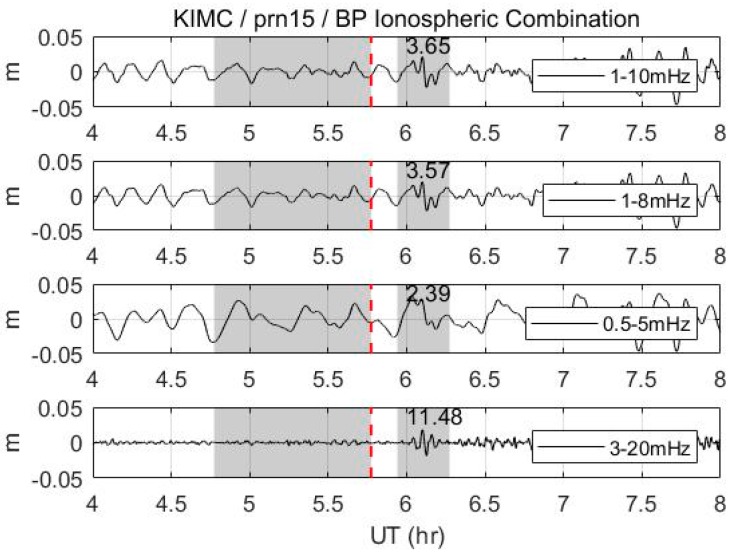
Band-passed ionospheric combination by different passbands (KIMC, prn15). Values in the right shade indicate the SNR. Refer to Figure 2 for notation.

Since previous studies, which used band-pass filters for de-trending, have not focused on enhancing the detection performance of the CID, and an appropriate passband-maximizing SNR is needed for a conservative comparison with the proposed algorithm. It is unrealistic, however, to optimize the band-pass filter for its complexity. We empirically observed that the 3–20 mHz passband would be the best option for maximizing the SNR of the CID for our datasets. In Figure 8, the values in the shade on the right indicate the SNR performance for each passband. It appears that the 3–20 mHz passband has a higher SNR performance than do the passbands that were used in previous studies. However, for rigorous study, further verification is needed on the relation between the passband and the SNR of the CID.

### 2.7. Applications for Early Detection Cases

**Figure 9:**
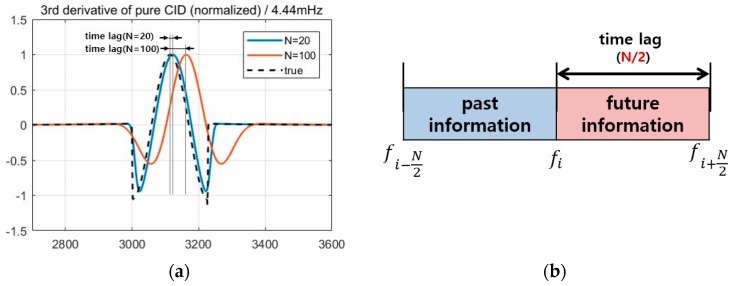
(**a**) The time lag that is inherent in the detection algorithms for the real-time application, and (**b**) its schematic.

Although the size of the maximum SNR is a major concern for the post-processing of data, there are other considerations for real-time applications. The detection algorithm uses a certain length of data before and after the epoch of interest, so it uses both past and future data for some particular time. Since future data are yet to be measured in real time, there exists a time lag that amounts to one half of N. Figure 9a shows simulated real-time ionospheric delay data with 4.44 mHz frequency, which accounts for one of the dominant normal frequencies between earthquakes and their atmospheric coupling [8]. The time lag with N=100 appears to be larger than that with N=20. For general cases, as illustrated in Figure 9b, the time lag in the real-time application is directly proportional to N.

There are real-time scenarios in which small time lags are required, such as with tsunami detection and early warning systems. In these scenarios, it is more beneficial to use proposed time derivative methods with a smaller value of N to enable early detection by allowing for a reduced time lag. In the next section, the performance of the proposed methods will be further discussed for smaller *N* values, to account for these real-time applications.

## 3. Results

### 3.1. Maximum SNR Comparisons

**Figure 10:**
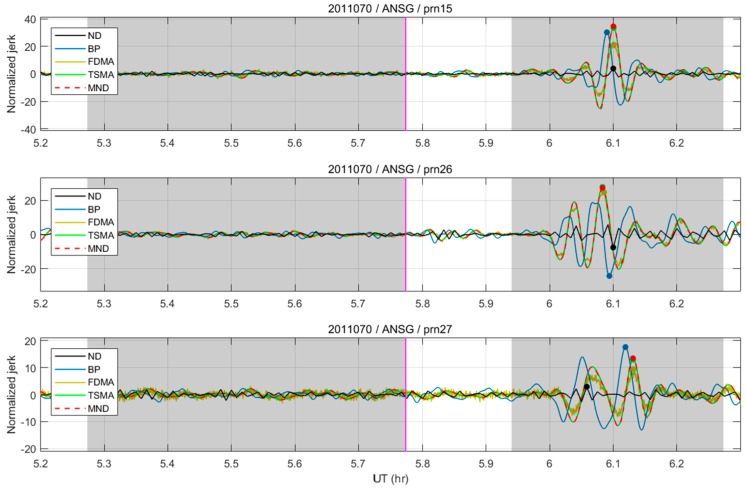
The filtering outputs of conventional (ND and BP) and proposed (FDMA, TSMA and MND) detection algorithms.

Figure 10 shows the filtering outputs of two conventional and three proposed algorithms. The “ND” indicates the conventional numerical third-order derivative with a 30-second interval [14,15]. In addition, “BP” stands for the band-pass filter with a 3–20 mHz passband, which gives a seemingly optimal SNR for this dataset. To assess the performance with a different PRN, the average of the SNR values at 45 reference stations is used, using the best *N* values from Table 2.

Table 3 shows the overall test results of the average values of the SNR for 45 NGII stations, using the best *N*s for each algorithm. PRN 26 shows the largest SNR due to the small distance of its IPP to the epicenter at the time of the earthquake. The MND showed a greater than 300% SNR improvement from the numerical third-order derivative, and a ~12–13% improvement from the band-pass filter and FDMA. In addition, it can be concluded from the results that the TSMA and MND attained similar performances, having a performance difference of less than 1%. In conclusion, the test results confirm that the TSMA and the MND provide a maximum detection performance of the CID.

### 3.2. SNR Comparisons in an Early Detection Case

**Figure 11:**
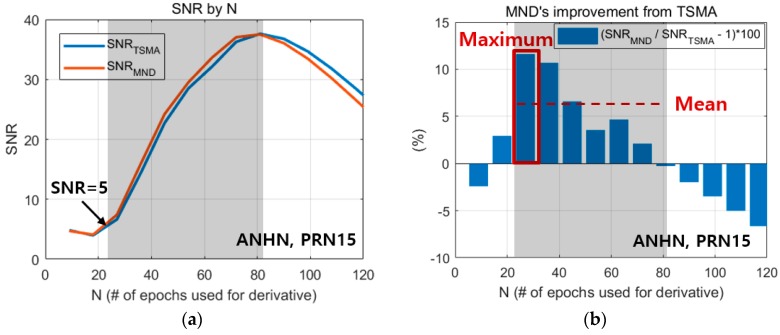
SNR comparison of the MND and the TSMA within the small-N region (shaded area) for the early detection case (ANHN station, PRN15). (**a**) SNR of the MND and the TSMA, (**b**) SNR improvement of the MND against the TSMA.

Figure 11a shows the SNR of the MND and TSMA according to N. Figure 11b shows the SNR improvement of the MND against the TSMA, especially in the small-*N* region. The small-N region is defined as the region where the SNR of the TSMA ranges from five to its maximum value, which is indicated by the shaded area in Figure 11. The minimum SNR value for determining the small-*N* region is set to five, because smaller SNR values, which correspond to signal amplitudes under $5\sigma_{noise}$, have the potential to cause frequent false alarms in real-time applications. In Figure 11b, MND shows a larger improvement percentage when N is 20 rather than 60, while the absolute SNR increase of MND from TSMA in Figure 11a is larger when N is 60. This is because in the small-N region, the SNR difference between MND and TSMA does not vary significantly according to N, while the absolute SNR values of both MND and TSMA increase drastically. Table 4 shows the maximum and mean values of the SNR improvements of the MND against the TSMA within the small-N region, where the averaged values for 45 NGII stations were used for analysis. The MND showed a ~6–7% SNR improvement in the maximum values, and a ~3–4% SNR improvement in the mean values, compared to those from the TSMA. Therefore, for real-time applications, the MND is recommended over the TSMA.

## 4. Conclusions

In this study, a novel time derivative method that can minimize noise levels was proposed and analyzed. By preserving disturbance signals and by reducing noise through the proposed method with their best N values, the CID can be effectively detected from the ionospheric combination. The suggested MND algorithm assures the minimum noise level under a couple of assumptions, and leads to enhanced detection performance. The SNR of the CID for the 2011 Tohoku earthquake data was maximized when estimating one epoch slope by using 100 sequential data points in 1-s interval data with the MND algorithm. SNR improvements of 12% and 13% were observed, compared to the FDMA and band-pass results, respectively. Also, the TSMA, which determines a moving average after the forward difference with an extended time interval, showed a similar performance to the MND method. However, for a small value of N, which enables fast detection and early warnings, the SNR of the MND is relatively higher than that of the TSMA. In conclusion, the MND would be the most effective solution in terms of both the detection performance and its application for early-warning cases. It should be noted that the derived best *N* in this article is most suitable for the 2011 Tohoku Earthquake event. For a more rigorous study, case studies of other earthquakes will be performed for future work.

## Figures and Tables

**Table 1:** The time-derivative equations and the noise levels of the conventional (FDMA) and the proposed (TSMA, MND) methods.

Algorithm	Time Derivative Equation	Noise Level
FDMA	${f^{\prime }}_{FDMA}=\frac{-f_{1}+f_{N}}{N-1}$	${\sigma^{\prime }}_{FDMA}=\frac{\sqrt{2}}{N-1}\sigma_{\nu }$
TSMA (proposed)	${f^{\prime }}_{TSMA}=\frac{-\left(f_{i}+\ldots +f_{i+K-1}\right)+\left(f_{N-K}+\ldots +f_{i+N-1}\right)}{K\left(N-K\right)}$	${\sigma^{\prime }}_{TSMA}=\frac{3\sqrt{6}}{2N\sqrt{N}}\sigma_{\nu }$
MND (proposed)	${f^{\prime }}_{MND}=c_{1}f_{i}+\ldots +c_{N}f_{i+N-1}$ where, $c_{k}=\frac{-6\left(N-1\right)+12\left(k-1\right)}{\left(N-1\right)N\left(N+1\right)}\;\;\left(k=1,2,\ldots ,N\right)$	${\sigma^{\prime }}_{MND}=\sqrt{\frac{12}{\left(N-1\right)N\left(N+1\right)}}\sigma_{\nu }$

**Table 2:** The best Ns for MND, FDMA, and TSMA.

Noise Reduction Method	Best N
MND 3rd	100
FDMA 3rd	80
TSMA 3rd	108

**Table 3:** Average values of the SNR for 45 NGII stations, using the best *N* values and SNR improvements of MND from other algorithms.

Algorithm	PRN 15	PRN 26	PRN 27	SNR Improvement of MND
Numerical 3rd (ND, 30-second)	3.20	8.92	2.25	340.6%
Band-pass (BP, 3–20 mHz)	16.65	20.08	8.05	13.6%
FDMA 3rd	14.27	28.40	6.88	12.5%
TSMA 3rd	17.71	29.37	7.95	−0.8%
MND 3rd	17.87	28.98	7.89	

**Table 4:** The SNR improvement of the MND against the TSMA within the small-N region (the average values of 45 NGII stations are used for analysis).

	Percentage Improvement (%)	
PRN	Maximum	Mean
15	7.4	3.8
26	5.9	2.2
27	5.9	2.7
